# Denture Plaque Biofilm Visual Assessment Methods: A Systematic Review

**DOI:** 10.1016/j.identj.2023.06.010

**Published:** 2023-07-19

**Authors:** Khaing Myat Thu, Andy Wai Kan Yeung, Lakshman Samaranayake, Walter Yu Hang Lam

**Affiliations:** Faculty of Dentistry, The University of Hong Kong, Hong Kong Special Administrative Region, People's Republic of China

**Keywords:** Denture hygiene, Denture cleanliness, Denture plaque, Plaque biofilm, indices, Planimetry

## Abstract

Denture plaque, a biofilm that develops on denture surfaces, could contribute to many oral and systemic afflictions. Hence, a quantitative assessment of denture plaque is important to evaluate the denture hygiene of denture wearers, particularly to prevent plaque biofilm-associated diseases. The aim of this systematic review, therefore, was to review and summarize the visual denture hygiene assessment methods using denture plaque indices and with planimetries published in the literature. English language studies published up to March 2022 in four electronic databases, PubMed, Medline, Embase, and Cochrane Library, were searched, followed by a manual search of Google Scholar by two assessors. The review followed Preferred Reporting Items for Systematic Reviews and Meta-Analyses (PRISMA) whenever possible. Details of the visual assessment methods, including the types of denture assessed, its materials and its surfaces, as well as the use of a disclosing agent, were the main outcomes. Of 492 screened studies, 74 were included per the inclusion and exclusion criteria. Of these, 60 studies utilized various denture plaque indices while 18 used planimetries. 43 out of 60 studies with indices and 17 out of 18 studies with planimetries used disclosing agents for visual evaluation of plaque. A total of 21 indices were described in the included studies, of which seven graded a divided denture surface, while the remainder graded entire denture surface. Of the 18 planimetric assessments, one study quantified squares of the disclosed plaques on denture images, 16 studies quantified such pixels with computer programs, and a single study quantified points, pixels, and contour of plaque areas. In summary, denture plaque indices appear to be popular in denture plaque assessment due to their simplicity. Computerized planimetric assessment, though more time-consuming, provides a more accurate assessment of plaque load as it is less prone to subjectivity and assessor errors.

## Introduction

Dental prostheses such as removable partial and complete dentures are still widely used to replace missing teeth, particularly in the developed world, mainly due to the higher financial outlay of various dental implants. The former exogenous, acrylic, or metallic, appliances are well known to harbor denture plaque biofilm in retentive areas, and these are likely to contribute to afflictions such as *Candida*-associated denture stomatitis, and caries and periodontitis of the abutment teeth.^1^, ^2^, ^3^, ^4^ Denture plaque is essentially a biofilm comprising a complex aggregate of micro-organisms and their metabolites that accumulate on denture surfaces.^2^

A positive correlation between the amount of denture plaque biofilm and the severity of denture stomatitis is well established.^5^^,^^6^ Furthermore, denture plaque aggregates particularly in debilitated hospitalized individuals are known to correlate well with potentially fatal systemic complications such as aspiration pneumonia.^1^^,^^7^^,^^8^ Meanwhile, most denture wearers, especially in developing countries, have poor knowledge of denture hygiene.^9^ Hence, over the years, many workers have attempted to either quantitatively or semi-quantitatively assess denture hygiene to educate and motivate patients in a visually impressive manner and also to develop clinical correlates of plaque-related diseases.^10^^,^^11^

The presence and distribution of plaque biofilm on the denture surfaces could be assessed using either the naked eye with denture plaque assessment indices or the latter with planimetries.^12^ Those indices utilize a calibrated assessor for semi-quantitative grading of denture plaque biofilm. The assessor usually assesses the area of denture surfaces covered with plaque biofilm using a disclosing agent.^12^ More recently, however, planimetries have been employed to provide a better quantitative assessment of plaque biofilm-covered denture surfaces. Computer programs are usually used in planimetric analyses to provide a quantitative indication of the area with plaque deposition. In general, denture plaque indices are also helpful to motivate individual patients to improve their denture hygiene while the latter, more sensitive planimetric assessment methods can be used in research settings to compare the relative efficacy of denture cleansing agents and methods.

Over the last few decades, many indices and planimetries evaluating denture hygiene for clinical as well as research purposes have been used and these have been reviewed in an attempt to obtain a global oversight on denture plaque levels,^2^ denture hygiene,^13^ and denture hygiene practices.^14^ However, to the best of our knowledge, there is no recent comprehensive review of the subject and the current review was undertaken to provide a contemporaneous, critical account of the visual denture hygiene assessment methods described in the literature.

## Methods

The review question “Which visual assessment methods have been used to measure denture plaque biofilm in previous studies?” was specifically set using a Population, Intervention, Control, and Outcomes (PICO) model (Table 1). This review was registered on the PROSPERO international prospective register of systematic reviews (CRD42023390370). The review followed the guidelines of Preferred Reporting Items for Systematic Reviews and Meta-Analyses (PRISMA).

**Table 1:** The search strategy and tools for risk of bias assessment used in the study

**Population**	Denture wearers
**Intervention**	Visual assessment methods of denture plaque-deposited area
**Comparison/ Control**	Comparison to clean or new dentures
**Outcomes**	Denture plaque deposited area
**Searched terms**	(“Denture hygiene” OR “denture cleanliness” OR “denture plaque” OR “denture care”) AND (“assessment” OR “observation” OR “study” OR “investigation” OR “score” OR “scale” OR “index” OR “method” OR “model” OR “way”)
**Database**	PubMed, Medline, Embase, and Cochrane Library
**Filter**	No filter applied
**Search date**	The most recent search was performed on March 31, 2022.
**Risk of Bias Assessment Tools**	The National Institutes of Health (NIH) quality assessment tools for controlled intervention studies/observational cohort and cross-sectional studies/before-after (Pre-Post) study with no control group/case-control studies/case series

### Literature Search

The literature in four electronic databases, PubMed, Medline, Embase, and Cochrane Library, was searched by two assessors (KMT and AWKY) independently using a defined search strategy (Table 1). Relevant references from the selected studies were retrieved and an additional hand-search via Google Scholar was performed by the same assessors to identify other potentially eligible studies. The most recent search was performed on March 31, 2022. Abovementioned two independent assessors initially screened through the titles and abstracts of retrieved studies. Duplicates of studies were removed. The shortlisted studies were then screened with full-text analysis. The inclusion criteria for this systematic review were as follows: English language studies; visual assessment of denture hygiene on removable partial or complete dentures; original clinical studies. Simulated *in vitro* studies, case reports and short communications, studies without statistical analysis as well as studies using assessment methods other than visual assessments were excluded. Disagreements between assessors were solved by discussion for a consensus.

### Data collection, extraction, and analysis

Data extraction was performed independently by the same assessors using a pre-defined data extraction template. Inter-assessor conflicts were discussed to reach a consensus. From the materials and methods section of each selected study, details of the denture hygiene assessment including the examined samples whether the actual denture or its images, complete or partial or both dentures, maxillary or mandibular or both dentures, types of denture materials, the use of a disclosing agent, and the assessed denture surfaces were extracted (Table 2 and 3). Furthermore, details of the denture plaque indices and planimetric assessment methods were also extracted (Table 4). Any missing information was secured as much as possible by emailing the corresponding author. The screening process and data extraction were performed using Covidence systematic review software (Veritas Health Innovation, Melbourne, Australia). The methodological quality of selected studies was assessed by an assessor (KMT) using National Institutes of Health (NIH) Study Quality Assessment Tools for respective study types.

**Table 2:** A list of the 74 reviewed studies. (*studies under the same project of Zenthofer et al. 2014^34^. ^#^studies under the same project of Sloane et al.^32^)

No.	Year	Authors	Content of study/ Purpose	Visual Assessment tools	Name of tools/studies used and referred	Examined sample (Denture/Image)	Profile of dentures assessed	Disclosing Materials (brands/ compound)	Denture surface(s) assessed
**1**	1970	Budtz-Jorgensen and Bertram^4^	Relationship between denture cleanliness and denture stomatitis	Index	Budtz-Jorgensen and Betram 1970^4^	Denture	Maxillary	Proflavine-monosulfate in 3.0 % aqueous solution	Fitting surface
**2**	1977	Budtz-Jorgensen and Kelstrup ^45^	The efficiency of denture cleansers (enzymes)	Index	Budtz-Jorgensen and Betram 1970^4^	Denture	Maxillary	Proflavine-monosulfate in 3.0 % aqueous solution	Fitting surface
**3**	1977	Budtz-Jorgensen^56^	Efficacy of enzymatic dissolvent tablet for prevention of denture plaque	Index	Budtz-Jorgensen 1977^56^	Image	Maxillary	Proflavine-monosulfate in 3.0 % aqueous solution	Fitting surface
**4**	1978	Budtz-Jorgensen and Knudsen^67^	Efficacy of brushing with chlorhexidine or Steradent for prevention of denture plaque	Index	Budtz-Jorgensen 1977^56^	Image	Maxillary	Proflavine-monosulfate in 3.0 % aqueous solution	Fitting surface
**5**	1981	Abelson et al.^78^	The efficiency of commercial denture cleansers	Index	Abelson et al.1981^78^	Denture	Any	Trace dye, The Lorvic Co. St. Louis, MO	All surfaces including teeth
**6**	1982	Ambjørnsen et al.^53^	Additive index for denture plaque accumulation	Index	Ambjørnsen et al. 1982^53^	Denture	Maxillary	No disclosing	Fitting surface
**7**	1982	Ghalichebaf et al.^68^	Effectiveness of commercial immersion-type denture cleansers	Planimetry-square counting	Square counting	Image	Maxillary	5.0% erythrosine	Fitting surface
**8**	1982	Tarbet et al.^83^	Relationship between denture hygiene and mucosal health	Index	Tarbet et al. 1982^83^	Denture	Maxillary	FD&C Red No. 3	Fitting surface
**9**	1982	Augsburger and Elahi^84^	Cleansing efficiency of soap-type denture cleanser	Index	Augsburger and Elahi 1982^84^	Denture	Maxillary	FD&C Red No. 3 (erythrosine) Lorric Corp., St. Louis, Mo.	All surfaces including teeth
**10**	1983	Budtz-Jorgensen et al.^15^	Efficacy of protease enzyme denture cleansers (Alcalase)	Index	Budtz-Jorgensen et al. 1983^15^	Image	Maxillary	Proflavine-monosulfate in 3.0 % aqueous solution	Fitting surface
**11**	1983	Poulsen et al.^16^	Evaluation of two methods of denture plaque scorings	Index	Budtz-Jorgensen et al. 1983^15^	Image	Maxillary	Proflavine-monosulfate in 3.0 % aqueous solution	Fitting surface
**12**	1984	Ambjørnsen et al.^17^	To compare the reproducibility and reliability of different denture plaque scorings	Index	Budtz-Jorgensen 1977^56^	Image	Maxillary	Proflavine-monosulfate in 3.0 % aqueous solution	Fitting surface
					Schubert and Schubert's PHI^86^				
					Ambjørnsen et al. 1982^53^				
**13**	1986	Murray et al.^18^	Relationship between the abrasivity and cleaning power of the dentifrices-type denture cleansers	Index	Murray et al. 1986^18^	Denture	Both	1.0% solution of fluorescein	All surfaces including teeth
**14**	1987	Schou et al.^54^	Relationship between oral hygiene, denture plaque, and stomatitis	Index	Armbjornsen et al. 1982^53^	Denture	Maxillary	No disclosing	Fitting surface
**15**	1990	Cardash et al.^19^	Method of monitoring denture hygiene	Index	Tarbet et al. 1982^83^	Image	Both	Red Cote, J.O. Butler. Co. Chicago III	Fitting surface
**16**	1990	Hoad-Reddick et al.^82^	Denture cleanliness in the elderly population	Index	Hoad-Reddick et al. 1990^82^	Denture	Both or any	No disclosing	All surfaces including teeth
**17**	1995	McCabe et al.^20^	Efficacy of two soaking denture cleansers	Index	McCabe et al. 1995^20^	Denture	Not mentioned	FDC blue 1, 0.25% in deionized water	All surfaces including teeth
**18**	1996	Jeganathan et al.^21^	Clinically viable denture hygiene index	Index	Jeganathan et al. 1996^21^	Denture	Maxillary	FD&C Red No. 3 (erythrosine)	Fitting surface
**19**	1996	McCabe et al.^22^	Method for denture plaque scoring	Index	McCabe et al. 1995^20^	Denture	Both	FDC blue 1, 0.25% in deionized water	All surfaces including teeth
**20**	1996	Keng and Lim^23^	Denture plaque distribution and effectiveness of a perborate-containing denture cleanser	Index	Modified Quigley-Hein Index^23^	Image	Both	Red Cote, J.O. Butler. Co. Chicago III	All surfaces including teeth
**21**	1997	Jeganathan et al.^24^	Relationship between denture hygiene habits, cleanliness, wearing behavior, and stomatitis	Index	Budtz-Jorgensen and Betram 1970^4^	Denture	Maxillary	FD&C Red No. 3 (erythrosine)	Fitting surface
**22**	2000	Sheen and Harrison^25^	A new method for assessing plaque levels on dentures by using digital imaging	Planimetry	Not mentioned the program used	Image	Maxillary	Fluorescent dye (Spectrum Chemical Mfg Corp, Gardena, Calif.)	All surfaces including teeth
				Index	Augsburger and Elahi 1982^84^	Denture	Maxillary		
**23**	2002	Kulak-Ozkan et al.^26^	Relationship between oral hygiene habits, denture cleanliness, and stomatitis	Index	Budtz-Jorgensen and Betram 1970^4^	Denture	Maxillary	Proflavine-monosulfate in 0.3% aqueous solution	Fitting surface
**24**	2004	Paranhos et al.^10^	Comparison of different denture plaque assessments methods	Planimetry	Image Tool 2.02 Software	Image	Maxillary	An aqueous solution of 5.0% erythrosine (Art. 1355 Erythrosine, E. Merck, Darmstadt, Germany)	Fitting surface
				Planimetry	Digital planimeter	Image	Maxillary		
				Planimetry-point counting	Grid with equidistant points	Image	Maxillary		
				Paper weighing	NOT visual method	-	-		
**25**	2004	Andrucioli et al.^69^	To evaluate the effectiveness of denture cleansing paste	Planimetry	Image Tool 2.02 Software	Image	Maxillary	1.0% sodium fluorescein	Fitting surface
**26**	2005	Kanli et al.^27^	Relationship between oral hygiene habits, denture cleanliness, and stomatitis	Index	Schubert and Schubert's PHI^86^	Denture	Maxillary	5.0% erythrosine dye solution	Fitting surface
**27**	2006	Montal et al.^55^	To assess oral (denture) hygiene, and treatment needs of the geriatric institution	Index	Montal et al. 2006^55^	Denture	Any	No disclosing	All surfaces including teeth
**28**	2006	De Visschere et al.^28^	Oral hygiene of the elderly in long-term care institutions	Index	Augsburger and Elahi 1982^84^	Denture	Any	Methylene blue disclosing solution	Fitting and polished surfaces
**29**	2006	Dikbas et al.^57^	Investigation of denture cleanliness	Index	Hoad-Reddick et al. 1990^82^	Denture	Both	No disclosing	All surfaces including teeth
**30**	2007	Fernandes et al.^70^	Comparison of efficacy of three denture brushes	Planimetry	Adobe Photoshop 5.5 software	Image	Both	An aqueous solution of 1.0% neutral red	All surfaces including teeth
**31**	2007	Paranhos et al.^29^	Distribution of biofilm on internal and external surfaces of the denture	Index	Paranhos et al. 2007^29^	Image	Maxillary	1.0% neutral red solution; School of Pharmaceutical Sciences, University of Sao Paulo, Brazil	All surfaces including teeth
**32**	2007	Paranhos et al.^30^	Effect of mechanical and chemical denture cleansing methods	Planimetry	Image tool 2.02 + Adobe Photoshop software 5.4	Image	Maxillary	1.0% neutral red solution; School of Pharmaceutical Sciences, University of Sao Paulo, Brazil	Fitting surface
				Index	Paranhos et al.2006				
**33**	2007	Salles et al.^71^	To compare and correlate denture plaque after brushing, associated with specific paste and soap	Planimetry	Image Tool 2.0 Software	Image	Not mentioned	1.0% neutral red solution; School of Pharmaceutical Sciences, University of Sao Paulo, Brazil	Fitting surface
**34**	2009	Coulthwaite et al.^12^	To compare currently available visual and planimetric plaque measurement	Index	McCabe et al. 1995^20^	Image	Both	Methylene blue disclosing solution (FDC Blue #1, 0.25% in deionized water)	All surfaces including teeth
				Planimetry	Adobe Photoshop (version 7; Adobe Systems Inc.)	Image			
				Index	Augsburger and Elahi 1982^84^	Image			
**35**	2010	Paranhos et al.^31^	Evaluation of three denture hygiene indices	Planimetry	Image Tool 2.02 Software	Image	Maxillary	5.0% erythrosine aqueous solution	Fitting surface
				Index	Schubert and Schubert's PHI^86^				
				Index	Jeganathan et al. 1996^21^				
				Index	Budtz-Jorgensen 1977^56^				
**36**	2010	Souza et al.^72^	Domestic use of disclosing solution for denture hygiene	Planimetry	Image Tool 2.02 Software	Image	Maxillary	1.0% neutral red solution; School of Pharmaceutical Sciences, University of Sao Paulo, Brazil	Fitting and polished surfaces
**37**	2011	Cruz et al.^73^	Effectiveness of chemical cleanser and ultrasonic device for denture hygiene	Planimetry	Image Tool 2.02 Software	Image	Maxillary	1.0% neutral red solution	Fitting surface
**38**	2012	Puskar et al.^58^	To examine the influence of gender, age, cleaning habits, and continuous wear of dentures on denture stomatitis	Index	Ambjørnsen et al. 1982^53^	Denture	Both	No disclosing	Fitting surface
**39**	2012	Taiwo et al.^59^	Denture hygiene of elderly	Index	Taiwo et al. 2012^59^	Denture	Not mentioned	No disclosing	Fitting surface
**40**	2012	de Andrade et al.^74^	Effect of Chlorhexidine on denture hygiene	Planimetry	Image Tool 3.0 Software	Image	Maxillary	1.0% neutral red solution	Fitting surface
**41**	2013	Sloane et al.^32^^#^	Effect of person-centered mouth care intervention	Index	Augsburger and Elahi 1982^84^	Denture	Any	Not mentioned the brand or compound	Fitting and polished surfaces
**42**	2014	Mylonas et al.^33^	Clinical audit in denture cleanliness	Index	Mylonas’ DCI^33^	Denture	Any	Plaqsearch, Malmö, Sweden	Fitting surface
**43**	2014	Zenthöfer et al.^34^*	Comparison of oral health and hygiene in patients with or without dementia	Index	Wefers’ DHI^85^	Denture	Both	Plaque Test; IvoclarVivadent, Schaan, Liechtenstein	All surfaces including teeth
**44**	2014	Zenthöfer et al.^35^*	Association of apraxia with oral hygiene	Index	Wefers’ DHI^85^	Denture	Both	Plaque Test; IvoclarVivadent, Schaan, Liechtenstein	All surfaces including teeth
**45**	2015	Almas et al.^36^	Simplified quantitative denture plaque index that	Index	Classification of Almas, Salameh, Kutkut, and Doubali-Denture Plaque Index (ASKD-DPI) ^36^	Image	Both	Diluted erythrosine solution (Red-Cote #28 red dye)	Fitting surface
**46**	2015	Khanagar et al.^37^	To assess the oral hygiene status of institutionalized dependent elders	Index	Augsburger and Elahi 1982^84^	Denture	Not mentioned	Plaque check disclosing solution	Fitting and polished surfaces
**47**	2016	Zenthöfer et al.^38^*	Improving the oral health of institutionalized dementia elders	Index	Wefers’ DHI^85^	Denture	Both	Plaque Test; IvoclarVivadent, Schaan, Liechtenstein	All surfaces including teeth
**48**	2016	Mylonas et al.^39^	Denture cleanliness of patients in a regional dental hospital	Index	Mylonas’ DCI^33^	Denture	Any	Plaqsearch, Malmö, Sweden	Fitting surface
**49**	2016	Steinmassl et al.^65^	Relationship of cognitive status to oral hygiene	Index	Wefers’ DHI^85^	Denture	Both	Not mentioned about disclosing	All surfaces including teeth
**50**	2016	Zenthöfer et al.^40^*	Effectiveness of carers’ education on oral health and denture hygiene improvements of dementia elders	Index	Wefers’ DHI^85^	Denture	Both	Plaque Test; IvoclarVivadent, Schaan, Liechtenstein	All surfaces including teeth
**51**	2016	Duyck et al.^41^	Impact of denture cleaning method and overnight storage condition on denture plaque	Index	Augsburger and Elahi 1982^84^	Denture	Mandibular	4.0% erythrosine disclosing solution	Fitting and polished surfaces
**52**	2016	Al-Kaisy et al.^75^	Assessment of denture hygiene habit	Planimetry	Image tool 2.02 software	Image	Maxillary	Methylene blue disclosing solution (FDC Blue #1, 0.25% in deionized water)	Fitting surface
**53**	2017	Zenthöfer et al.^42^*	Association of dementia with poor oral health/denture hygiene and risk of periodontal disease in elderly	Index	Wefers’ DHI^85^	Denture	Both	Plaque Test; IvoclarVivadent, Schaan, Liechtenstein	All surfaces including teeth
**54**	2017	Nihtila et al.^60^	Effectiveness of a tailored oral health intervention	Index/ Score	Binary score (Y/N)	Denture	Not mentioned	No disclosing	Not mentioned
**55**	2017	Martori et al.^43^	Relationship between denture-related factors and oral Candida colonization	Index	Jeganathan et al. 1996^21^	Denture	Maxillary	Erythrosine (Reveal; Henry-Schein, Melville, NY	Fitting surface
**56**	2017	Zimmerman et al.^44^^#^	Oral hygiene status and risk assessment	Index	Augsburger and Elahi 1982^84^	Denture	Any	Not mentioned the brand or compound	Fitting and polished surfaces
**57**	2017	Arruda et al.^76^	Efficacy of denture cleanser	Planimetry	Image tool software	Image	Both	1.0% neutral red	Fitting surface
**58**	2018	Baba et al.^77^	To evaluate mechanical cleansing vs mechanical + chemical cleansing	Planimetry	Image J software	Image	Maxillary	Prospec, GC Co.	Fitting surface
**59**	2018	Ikeya et al.^79^	Effects of denture cleanser	Planimetry	Adobe Photoshop vCS6 software; Adobe Systems, Inc	Image	Maxillary	Methylene blue, 0.25% m/v in distilled water; Wako Pure Chemical Industries Ltd	All surfaces including teeth
**60**	2018	Schwindling et al.^46^*	Oral health intervention and denture hygiene	Index	Wefers’ DHI^85^	Denture	Any	Not mentioned about disclosing	All surfaces including teeth
**61**	2018	Klotz et al.^47^*	Oral health on the mortality of elderly people	Index	Wefers’ DHI^85^	Denture	Not mentioned	Plaque Test; IvoclarVivadent, Schaan, Liechtenstein	All surfaces including teeth
**62**	2018	Guevara-Canales et al.^61^	To determine whether self-perceived oral health impact and satisfaction measure	Index	Guevara-Canales et al.2018^61^	Denture	Both or any	No disclosing	Not mentioned
**63**	2018	Weintraub et al.^48^^#^	Improving oral hygiene in the nursing home	Index	Augsburger and Elahi 1982^84^	Denture	Both	Not mentioned the brand or compound	All surfaces including teeth
**64**	2020	Klotz et al.^49^*	To identify how changes to general health might affect the oral health	Index	Wefers’ DHI^85^	Denture	Not mentioned	Plaque Test; IvoclarVivadent, Schaan, Liechtenstein	All surfaces including teeth
**65**	2020	Badaró et al.^80^	Effects of three denture disinfection protocols	Planimetry	ImageTool 3.0; Informer Technologies, Inc	Image	Maxillary	No disclosing	Fitting surface
**66**	2020	Krausch-Hofmann et al.^11^	Assessment of oral health conditions presented in the photograph	Index	Krausch-Hofmann et al. 2020^11^	Denture	Maxillary	No disclosing	Fitting surface
**67**	2021	Alqarni et al.^50^	To analyze the influence of the intervention on neglected elderly	Index	Jeganathan et al. 1996^21^	Denture	Maxillary	Not mentioned about disclosing	Fitting surface
**68**	2021	Garg et al.^51^	Impact of Sensitization on Family Caregivers	Index	Jeganathan et al. 1996^21^	Denture	Both or any	Not mentioned the brand or compound	Not mentioned
**69**	2021	Ng et al.^52^	Effect of educational mobile application on denture hygiene	Index	Jeganathan et al. 1996^21^	Image	Maxillary	GC Tri Plaque ID Gel plaque disclosing agent (GC Co.)	Fitting surface
**70**	2021	Cinquanta et al.^62^	Patient attitude and habits on denture hygiene	Index	Hoad-Reddick et al. 1990^82^	Denture	Both	No disclosing	All surfaces including teeth
**71**	2021	Wiatrak et al.^66^	Effect of Tea Tree Oil Toothpaste on oral health	Index	Not mentioned	Not mentioned	Not mentioned	Not mentioned about disclosing	Not mentioned
**72**	2021	Araujo et al.^81^	Effect of denture hygiene protocol	Planimetry	NIS-elements software	Image	Maxillary	1.0% neutral red; IMBRALAB Química e Farmacêutica Ltda	Fitting surface
**73**	2022	Mousa et al.^63^	Development of halitosis	Index	Ambjørgensen et al. 1982^53^	Denture	Both	No disclosing	Fitting and polished surfaces
**74**	2022	Peroz et al.^64^	The influences of quarterly professional dental hygiene interventions	Index	Wefers’ DHI^85^	Denture	Any	No disclosing	All surfaces including teeth

**Table 3:** A summary of the included visual assessment of denture hygiene studies. Four studies used both assessment methods and are hence mutually inclusive in both categories.

	Studies using denture plaque indices		Studies using planimetries	
**Numbers of studies**	60	81.1%	18	24.3%
**Examined samples**				
Actual dentures	46	76.7%	0	0%
Images	13	21.7%	18	100%
Not mentioned	1	1.6%	0	0%
**Types of dentures assessed**				
Complete Denture	8	13.3%	10	55.6%
Partial Denture	2	3.3%	0	0%
Both complete and partial dentures	3	5.0%	0	0%
Not mentioned	47	78.3%	8	44.4%
**Profile of dentures assessed**				
Maxillary dentures	23	38.3%	14	77.8%
Mandibular dentures	1	1.7%	0	0%
Both maxillary and mandibular dentures	17	28.3%	3	16.6%
Maxillary or mandibular dentures	12	2.0%	0	0%
Not mentioned	7	11.7%	1	5.6%
**Materials of denture assessed**				
Acrylic	13	21.7%	10	55.6%
Metal	1	1.7%	0	0%
Both	1	1.7%	0	0%
Not mentioned	45	75.0%	8	44.4%
**Using of disclosing agents on examined samples**				
Yes	43	71.6%	17	94.4%
No (Plain)	13	21.7%	1	5.6%
Not mentioned	4	6.7%	0	0%
**Denture surface(s) assessed**				
Fitting (intaglio) surface only	26	43.3%	13	72.2%
Polished (cameo) surface only	6	1.0%	1	5.6%
All denture surfaces	24	40.0%	4	22.2%
Not mentioned	4	6.7%	0	0%

**Table 4:** Details about different denture plaque assessment indices and planimetric methods utilized to measure denture hygiene in the reviewed studies

Year	Name of Indices/ Planimetric methods	Examined sample (Denture/Image)	Profile of dentures assessed	Disclosing (Yes/No)	Denture surface(s) assessed	Entire surface or divided assessment	Grading method on denture plaque or denture cleanliness
**DENTURE PLAQUE INDICES**							
**1970**	Budtz-Jorgensen and Betram^4^	Denture	Maxillary	Yes	Fitting surface	Entire	Estimated proportion/ quality
**1977**	Budtz-Jorgensen^56^	Image	Maxillary	Yes	Fitting surface	Entire	Estimated proportion/ quality
**1979**	Schübert and Schübert Prosthesis Hygiene Index (PHI)^86^	Image	Maxillary	Yes	Fitting surface	Divided	Estimated proportion/ quality
**1981**	Abelson et al.^78^	Denture	Any	Yes	All surfaces including teeth	Entire	Estimated proportion/ quality
**1982**	Ambjørnsen et al.^53^ (developed from Silness and Loe plaque score 1964, Ainamo and Bay 1975)	Denture	Maxillary	No	Fitting surface	Divided	Estimated proportion/ quality
**1982**	Tarbet et al.^83^	Image	Both	Yes	Fitting surface	Divided	Estimated area %
**1982**	Augsburger and Elahi^84^	Denture	Maxillary	Yes	All surfaces	Divided	Estimated area %
**1983**	Budtz-Jorgensen et al.^15^	Image	Maxillary	Yes	Fitting surface	Entire	Estimated area %
**1984**	Modified Quigley-Hein Index ^23^	Denture	Maxillary	Yes	All surfaces including teeth	Entire	Estimated area %
**1986**	Murray et al.^18^	Denture	Both	Yes	All surfaces including teeth	Entire	Estimated proportion/ quality
**1990**	Hoad-Reddick et al.^82^	Denture	Both	No	All surfaces including teeth	Entire	Estimated proportion/ quality
**1995**	McCabe et al.^20^	Denture	Not mentioned	Yes	All surfaces including teeth	Entire	Estimated proportion/ quality (upon stain, soil calculus, disclosed plaque)
**1996**	Jeganathan et al.^21^ (modified Tarbet et al.)	Denture	Maxillary	Yes	Fitting surface	Entire	Estimated area %
**1999**	Wefers' Denture Hygiene Index (DHI)^85^	Denture	Both	Yes	All surfaces including teeth	Divided	Approximate % (the ratio of plaque-positive sites to all available sites, expressed as a percentage)
**2006**	Montal et al.^55^	Denture	Any	No	All surfaces including teeth	Entire	Estimated proportion/ quality
**2007**	Paranhos et al.^29^ (modified **Schübert and Schübert** PHI)	Image	Maxillary	Yes	All surfaces including teeth	Divided	Estimated proportion/ quality
**2012**	Taiwo et al.^59^ (modified WHO assessment 1986)	Denture	Any	No	Fitting surface	Entire	Estimated proportion/ quality
**2014**	Mylonas et al. Denture Cleanliness Index (DCI)^33^	Denture	Any	Yes	Fitting surface	Entire	Estimated area %
**2015**	Classification of Almas, Salameh, Kutkut, and Doubali-Denture Plaque Index (ASKD-DPI)^36^	Image	Both	Yes	Fitting surface	Divided	Estimated area %
**2018**	Guevara-Canales et al.^61^	Denture	Not mentioned	No	Not mentioned	Entire	Estimated proportion/ quality
**2020**	Krausch-Hofmann et al.^11^	Denture	Maxillary	No	Fitting surface	Entire	Estimated proportion/ quality
**PLANIMETRIC METHODS**							
	Computerized pixel-counting	Image	Maxillary	Yes	Fitting surface	Entire	Pixels of disclosed plaque area can be automatically counted by the image analysis software.
	Point-counting	Image	Maxillary	Yes	Fitting surface	Entire	Disclosed denture image was projected, and superimposed by a grit of squares or equidistant points. Percentage of denture areas with disclosed squares/points could be calculated.
	Square-counting	Image	Maxillary	Yes	Fitting surface	Entire	
	Digital planimeter	Image	Maxillary	Yes	Fitting surface	Entire	A digital planimeter traced the contour of disclosed plaque area and the entire denture surface to calculate the percentage of disclosed plaque area.

## Results

**Figure 1:**
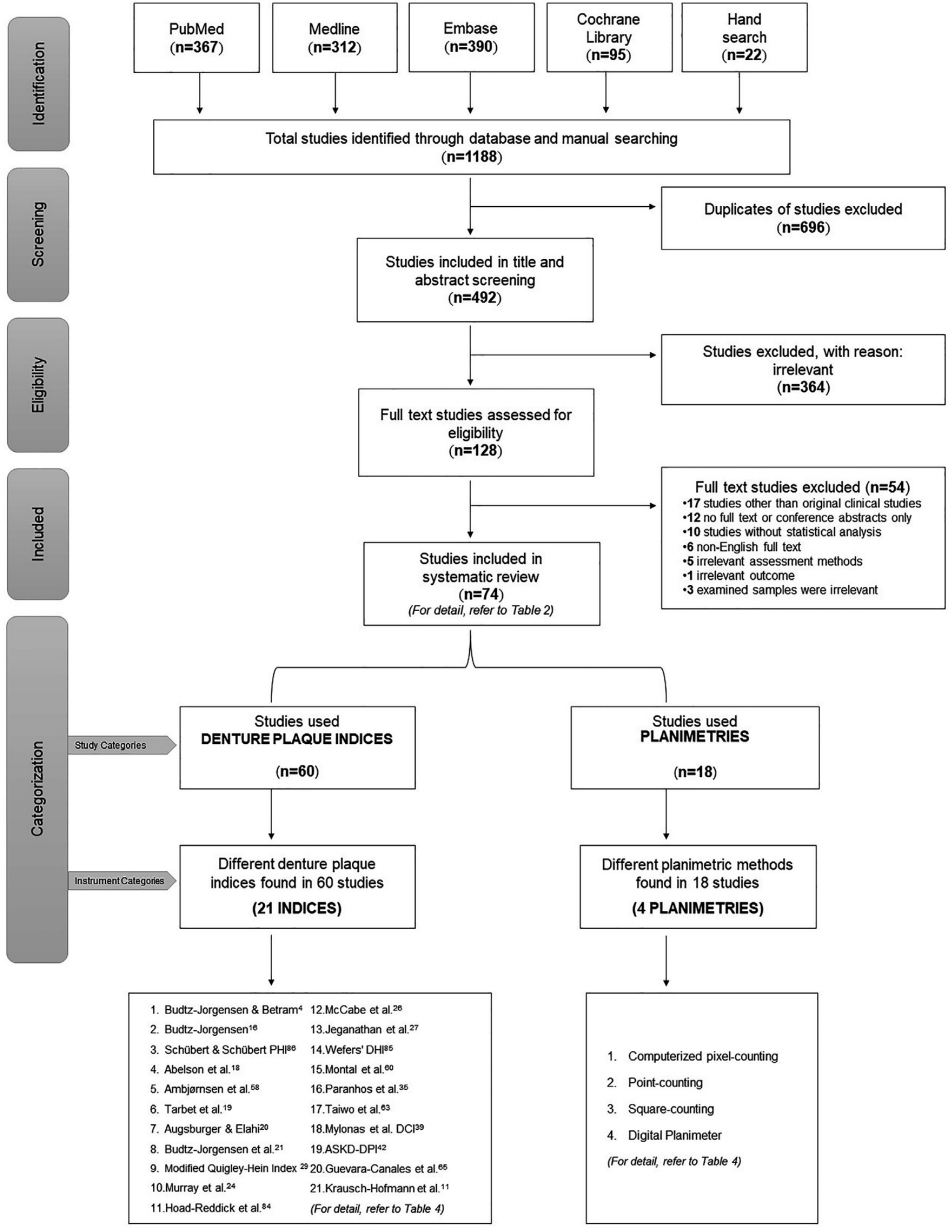
PRISMA flowchart for search strategy together with the structure of this systematic review. (Four studies used both assessment methods and are hence mutually inclusive in both categories.)

A total of 1188 studies were retrieved through the primary literature search. After the removal of duplicates and other exclusion criteria, 492 studies were screened, and of these, 128 were shortlisted for inclusion based on the screening of their titles and abstracts. The full text of shortlisted studies was assessed for eligibility, and finally, 74 studies^4^^,^^10^, ^11^, ^12^^,^^15^, ^16^, ^17^, ^18^, ^19^, ^20^, ^21^, ^22^, ^23^, ^24^, ^25^, ^26^, ^27^, ^28^, ^29^, ^30^, ^31^, ^32^, ^33^, ^34^, ^35^, ^36^, ^37^, ^38^, ^39^, ^40^, ^41^, ^42^, ^43^, ^44^, ^45^, ^46^, ^47^, ^48^, ^49^, ^50^, ^51^, ^52^, ^53^, ^54^, ^55^, ^56^, ^57^, ^58^, ^59^, ^60^, ^61^, ^62^, ^63^, ^64^, ^65^, ^66^, ^67^, ^68^, ^69^, ^70^, ^71^, ^72^, ^73^, ^74^, ^75^, ^76^, ^77^, ^78^, ^79^, ^80^, ^81^, ^82^, ^83^, ^84^ were selected for this review (Fig 1). A summary of all reviewed studies is listed in Table 2.

An overwhelming, 19 studies^12^^,^^20^^,^^21^^,^^25^^,^^29^, ^30^, ^31^^,^^33^^,^^39^^,^^41^^,^^63^^,^^66^^,^^73^, ^74^, ^75^, ^76^^,^^79^^,^^81^^,^^84^ assessed acrylic dentures, and only a single study^52^ investigated metallic dentures, whereas another^43^ investigated both acrylic and metallic dentures; the remaining 53 studies^4^^,^^9^^,^^10^^,^^14^, ^15^, ^16^, ^17^, ^18^^,^^21^, ^22^, ^23^^,^^25^, ^26^, ^27^^,^^31^^,^^33^, ^34^, ^35^, ^36^, ^37^^,^^39^^,^^41^^,^^43^, ^44^, ^45^, ^46^, ^47^, ^48^, ^49^^,^^50^^,^^52^, ^53^, ^54^, ^55^, ^56^, ^57^, ^58^, ^59^^,^^60^^,^^61^^,^^63^^,^^64^^,^^66^, ^67^, ^68^, ^69^^,^^70^^,^^71^^,^^76^^,^^77^^,^^79^^,^^81^^,^^82^ did not specify the fabricated denture material under investigation. (Table 3) Surprisingly, eight^34^^,^^35^^,^^38^^,^^40^^,^^42^^,^^46^^,^^47^^,^^49^ of 74 studies reported results from the identical population by a single group of investigators in Germany, and three studies^32^^,^^44^^,^^48^ investigated another single cohort from the USA. (Table 2)

The totally included 74 studies were split into two sub-categories as per the assessment methods. Accordingly, 18 studies^10^^,^^12^^,^^25^^,^^30^^,^^31^^,^^68^, ^69^, ^70^, ^71^, ^72^, ^73^, ^74^, ^75^, ^76^, ^77^^,^^79^, ^80^, ^81^ used planimetries while 60 studies^4^^,^^11^^,^^12^^,^^15^, ^16^, ^17^, ^18^, ^19^, ^20^, ^21^, ^22^, ^23^, ^24^, ^25^, ^26^, ^27^, ^28^, ^29^, ^30^, ^31^, ^32^, ^33^, ^34^, ^35^, ^36^, ^37^, ^38^, ^39^, ^40^, ^41^, ^42^, ^43^, ^44^, ^45^, ^46^, ^47^, ^48^, ^49^, ^50^, ^51^, ^52^, ^53^, ^54^, ^55^, ^56^, ^57^, ^58^, ^59^, ^60^, ^61^, ^62^, ^63^, ^64^, ^65^, ^66^, ^67^^,^^78^^,^^82^, ^83^, ^84^ used denture plaque indices (Table 3 and Fig 1). Four studies^12^^,^^25^^,^^30^^,^^31^ used both assessment methods, hence such studies were mutually inclusive in both categories. Therefore, the total sum of studies of all categories evaluated was over 74. All but one^81^ of the included studies had a moderate to high risk of bias (Supplementary Table 1).

### Characteristics for included studies that utilized denture plaque indices

Of the 60 studies using denture plaque indices with naked eye assessment,^4^^,^^11^^,^^12^^,^^15^, ^16^, ^17^, ^18^, ^19^, ^20^, ^21^, ^22^, ^23^, ^24^, ^25^, ^26^, ^27^, ^28^, ^29^, ^30^, ^31^, ^32^, ^33^, ^34^, ^35^, ^36^, ^37^, ^38^, ^39^, ^40^, ^41^, ^42^, ^43^, ^44^, ^45^, ^46^, ^47^, ^48^, ^49^, ^50^, ^51^, ^52^, ^53^, ^54^, ^55^, ^56^, ^57^, ^58^, ^59^, ^60^, ^61^, ^62^, ^63^, ^64^, ^65^, ^66^, ^67^^,^^78^^,^^82^, ^83^, ^84^ a vast majority 46 assessed the actual dentures, ^4^^,^^11^^,^^18^^,^^20^, ^21^, ^22^^,^^24^, ^25^, ^26^, ^27^, ^28^^,^^32^, ^33^, ^34^, ^35^^,^^37^, ^38^, ^39^, ^40^, ^41^, ^42^, ^43^, ^44^, ^45^, ^46^, ^47^, ^48^, ^49^, ^50^, ^51^^,^^53^, ^54^, ^55^^,^^57^, ^58^, ^59^, ^60^, ^61^, ^62^, ^63^, ^64^, ^65^^,^^78^^,^^82^, ^83^, ^84^ while 13 assessed only the denture images.^12^^,^^15^, ^16^, ^17^^,^^19^^,^^23^^,^^29^, ^30^, ^31^^,^^36^^,^^52^^,^^56^^,^^67^ One study did not mention the assessed item.^66^ Twenty-three studies examined the maxillary dentures only,^4^^,^^11^^,^^15^, ^16^, ^17^^,^^21^^,^^24^, ^25^, ^26^, ^27^^,^^29^, ^30^, ^31^^,^^43^^,^^45^^,^^50^^,^^52^, ^53^, ^54^^,^^56^^,^^67^^,^^83^^,^^84^ 17 studies examined both the maxillary and mandibular dentures,^12^^,^^18^^,^^19^^,^^22^^,^^23^^,^^34^, ^35^, ^36^^,^^38^^,^^40^^,^^42^^,^^48^^,^^57^^,^^58^^,^^62^^,^^63^^,^^65^ and 12 studies examined either the maxillary or the mandibular dentures.^28^^,^^32^^,^^39^^,^^44^^,^^46^^,^^51^^,^^55^^,^^61^^,^^64^^,^^74^^,^^78^^,^^82^ Studies that used disclosing agents to assess denture plaque (43 studies)^4^^,^^12^^,^^15^, ^16^, ^17^, ^18^, ^19^, ^20^, ^21^, ^22^, ^23^, ^24^, ^25^, ^26^, ^27^, ^28^, ^29^, ^30^, ^31^, ^32^, ^33^, ^34^, ^35^, ^36^, ^37^, ^38^, ^39^, ^40^, ^41^, ^42^, ^43^, ^44^, ^45^^,^^47^, ^48^, ^49^^,^^51^^,^^52^^,^^56^^,^^67^^,^^78^^,^^83^^,^^84^ were three times more than those without disclosing agents (13 studies).^11^^,^^20^^,^^53^, ^54^, ^55^^,^^57^, ^58^, ^59^, ^60^, ^61^, ^62^, ^63^, ^64^

The fitting surface of dentures was assessed in 26 studies^4^^,^^11^^,^^15^, ^16^, ^17^^,^^19^^,^^24^^,^^26^^,^^27^^,^^30^, ^31^, ^32^^,^^36^^,^^39^^,^^43^^,^^45^^,^^49^^,^^50^^,^^52^, ^53^, ^54^^,^^56^^,^^58^^,^^59^^,^^67^^,^^83^ while both the denture and tooth surfaces were assessed in 24 studies.^12^^,^^18^^,^^20^^,^^22^^,^^23^^,^^25^^,^^29^^,^^34^^,^^35^^,^^38^^,^^40^^,^^42^^,^^46^, ^47^, ^48^, ^49^^,^^55^^,^^57^^,^^62^^,^^64^^,^^65^^,^^78^^,^^82^^,^^84^ The remaining six studies assessed only the fitting and polished surfaces of dentures without teeth,^28^^,^^32^^,^^37^^,^^41^^,^^44^^,^^63^ and four studies did not mention the surface(s) they assessed. ^51^^,^^60^^,^^61^^,^^66^

### Characteristics for included denture plaque indices

In total, 21 indices were used for denture plaque grading by the naked eye of assessors (Table 4). The most commonly used indices were Wefers’ Denture Hygiene Index (DHI)^85^ which was used in 10 studies,^34^^,^^35^^,^^38^^,^^40^^,^^42^^,^^46^^,^^47^^,^^49^^,^^64^^,^^65^ and Augsburger and Elahi index which was used in nine studies.^12^^,^^25^^,^^28^^,^^32^^,^^37^^,^^41^^,^^44^^,^^48^^,^^84^

Disclosing agents for the denture plaque were utilized in 15 indices^4^^,^^15^^,^^18^^,^^20^^,^^21^^,^^23^^,^^29^^,^^33^^,^^36^^,^^56^^,^^78^^,^^83^, ^84^, ^85^, ^86^ while the remaining six indices did not utilize any disclosing agents.^11^^,^^53^^,^^55^^,^^59^^,^^61^^,^^82^ Ten indices were used to assess maxillary complete dentures,^4^^,^^11^^,^^15^^,^^21^^,^^23^^,^^29^^,^^53^^,^^56^^,^^84^^,^^86^ and nine for evaluation of maxillary and mandibular dentures.^18^^,^^33^^,^^36^^,^^55^^,^^59^^,^^78^^,^^82^^,^^83^^,^^85^ Two indices did not specify the type of examined dentures.^20^^,^^61^ Eight indices assessed all denture surfaces including denture teeth,^18^^,^^20^^,^^23^^,^^29^^,^^55^^,^^78^^,^^82^^,^^85^ one index assessed fitting (intaglio) and polished (cameo) surfaces;^84^ 11 indices assessed denture fitting surface only,^4^^,^^11^^,^^15^^,^^21^^,^^33^^,^^36^^,^^53^^,^^56^^,^^59^^,^^83^^,^^86^ and one did not specify the surface(s) assessed.^61^

In general, grading indices could be classified into three types: i) subjective grading by estimating the percentage of plaque-deposited areas (7 indices),^15^^,^^21^^,^^23^^,^^33^^,^^36^^,^^83^^,^^84^ ii) subjective grading by estimating the proportion and quality of plaque-deposited areas (13 indices),^4^^,^^11^^,^^18^^,^^29^^,^^53^^,^^55^^,^^56^^,^^59^^,^^61^^,^^78^^,^^82^^,^^86^ and iii) sub-dividing the denture surfaces into areas, and quantifying the percentage of divided areas with plaque deposition (1 index).^85^

Seven indices divided the denture surface into areas for grading,^29^^,^^36^^,^^53^^,^^83^, ^84^, ^85^, ^86^ while the rest graded the entire denture area, mostly the fitting surface.^4^^,^^11^^,^^15^^,^^18^^,^^20^^,^^21^^,^^23^^,^^33^^,^^55^^,^^56^^,^^59^^,^^61^^,^^78^^,^^82^ The index proposed by Jeganathan et al.^21^ was used to assess metallic dentures in two studies.^43^^,^^52^

### Planimetric assessments

Most planimetric studies assessed the fitting surface of the maxillary complete dentures. All except one planimetric assessment^80^ assessed the disclosed denture images i.e. 17 studies.^10^^,^^12^^,^^25^^,^^30^^,^^31^^,^^68^, ^69^, ^70^, ^71^, ^72^, ^73^, ^74^, ^75^, ^76^, ^77^^,^^79^^,^^81^ Of the 18 studies that utilized planimetric assessments, one study quantified squares,^68^ 16 studies quantified pixels with computer programs,^12^^,^^25^^,^^30^^,^^31^^,^^69^, ^70^, ^71^, ^72^, ^73^, ^74^, ^75^, ^76^, ^77^^,^^79^, ^80^, ^81^ and a single study quantified points, pixels, and contour of plaque areas.^10^ (Table 4) Among 17 studies that utilized computerized planimetries,^10^^,^^12^^,^^25^^,^^30^^,^^31^^,^^69^, ^70^, ^71^, ^72^, ^73^, ^74^, ^75^, ^76^, ^77^^,^^79^, ^80^, ^81^ two most commonly used software were UTHSCSA (the University of Texas Health Science Center at San Antonio) Image tool (11 studies)^10^^,^^30^^,^^31^^,^^69^^,^^71^, ^72^, ^73^, ^74^, ^75^, ^76^^,^^80^ and Adobe Photoshop (4 studies).^12^^,^^30^^,^^70^^,^^79^

### Comparative studies

One study compared the accuracy and reproducibility of the three indices (Schübert and Schübert Prosthesis Hygiene Index (PHI),^86^ Budtz-Jorgensen^56^ and Jeganathan et al.^21^) using the planimetric assessment as the gold standard.^31^ Another study analyzed the agreements between an index of Augsburger and Elahi^84^ and three planimetries.^12^

Since these various studies used disparate indices yielding different outcome parameters, a meta-analysis of the extracted data could not be performed.

## Discussion

In general, denture hygiene or denture plaque biofilm can be visually assessed using various indices and planimetries. Our review provides a contemporaneous account of these assessment methods described in the literature up to 2022. Clearly, the fact that there are so many methodologies in use to evaluate denture plaque biofilm implies that there is no single preferred method of assessment, and the data from the current review should facilitate decision-making by future investigators and clinicians embarking on similar studies on the optimal method of denture plaque evaluation.

In this review, most studies assessed acrylic complete dentures. Only a relatively small number of studies investigated metallic dentures and this may be related to imaging issues and poor contrast of disclosed metallic surfaces that may interfere with the computerized assessment of images.

Denture plaque biofilm usually develops unevenly on denture surfaces and more biofilm growth is seen on the fitting (intaglio) surface than on the polished (cameo) surfaces.^11^^,^^79^ This is because the intaglio surfaces are protected from the continuous, dynamic flushing action of saliva and the muscular movements of the tongue.^6^ Additionally, the intaglio surfaces, in comparison to cameo surfaces, are unpolished and may contain undercut regions, especially at the area around maxillary tuberosities and palatal rugae^19^^,^^30^ which have limited access for a denture brush.^79^ This, together with the fact that the maxillary denture-bearing area is the main plaque-depository area,^12^ and most affected by pathologies such as denture-associated stomatitis and related fungal infections.^87^ These are the possible reasons why most workers have assessed the intaglio surfaces of maxillary dentures in comparison to cameo surfaces. Actually, indices that can effectively assess both maxillary and mandibular dentures should be preferable clinically as many patients have both maxillary and mandibular prostheses. Besides, the differences in plaque patterns between maxillary vs mandibular dentures were not clearly reported in reviewed studies. Moreover, there was no report of a significant difference in plaque score or plaque amount between maxillary and mandibular dentures.

Furthermore, because of different plaque growth on different denture surfaces, plaque assessment on divided areas enables assessment of localized areas of plaque deposition.^17^ Of the indices used, the method of Augsburger and Elahi^84^ where eight sub-divided areas were assessed is clearly less time-consuming^12^ and preferable to the method of Paranhos et al.^29^ where a total of 22 sub-divided areas were quantified.

Most denture plaque assessment indices were inexpensive and simple to use, being compatible with use in a clinic or community setting, thus permitting the study of a large number of subjects quickly and effectively.^88^ The ease and the rapidity of the assessment method are clearly important in community-level studies with large cohorts, as visual fatigue associated with prolonged assessment^36^ could bias the outcome. Actually, assessment of actual dentures is quicker and simpler to grade for the entire surfaces, and also possible to use a blunt probe to physically detect the plaque^53^^,^^54^^,^^59^ but assessors can easily confuse with any imaginary division of the denture surfaces in the assessment on divided denture-areas. Hence assessments of the denture images, captured by a camera, rather than on-site evaluation, have been suggested so that assessors evaluate the images at a later stage in a laboratory setting with no time constraints.^20^^,^^22^ Furthermore, denture images can be anonymized easily in this manner to reduce any potential evaluator bias. The use of such imaging also facilitates the testing and training of inter- and intra-assessor reproducibility as well as allows dividing denture surface into areas by computer. Thus the suggested imaging techniques are in general preferable to on-site naked-eye evaluations.

Disclosing the plaque on the denture surfaces is demanding because plaque is usually colorless and cannot be visible easily.^89^^,^^90^ Disclosing agents are surrogate visual indicators^19^ that are used in many denture plaque indices (15 out of 21) in this review to enhance the visibility of plaque biofilm. The dye in the disclosing agent diffuses into plaque, binds to plaque components such as proteins and polysaccharides, and is retained in the plaque.^91^^,^^92^ All but a single denture plaque index^85^ in our review were entailing subjective judgments by the assessors and hence more prone to inter- and intra-assessor measurement errors. Moreover, these indices were in the ordinal scale, which is non-continuous and semi-quantitative, meaning that only low-power categorical statistical tests could be performed.^12^ An index that calculates the percentage of divided areas with plaque-deposited, is more objective and permits the use of powerful statistical tests, which is preferable in this context.^85^

Planimetric plaque assessment methods, as opposed to the traditional naked eye methods with indices, could be considered as a relatively new development in denture plaque evaluation research.^29^ In principle, these methods are based on quantifying^10^ either point counts or divided squares^68^ of a projected image or tracing the contour of a disclosed plaque area using a digital planimeter.^10^ Then, the area with plaque deposition, in pixels, can be automatically measured using image analysis software.

As regards the surfaces studied in planimetric investigations most evaluated only the fitting surface while a few others assessed multiple surfaces such as both the intaglio and cameo surfaces, and left and right buccal surfaces.^12^^,^^71^^,^^79^ Such planimetric assessment of multiple denture surfaces is useful for evaluating the efficacy of denture cleansing procedures though it may lead to confusion as overlapping image surfaces in different images.

The other advantage of planimetries is that the results are provided in percentages (%), as a continuous numerical value i.e. ratio scale^93^ that permits more powerful statistical analyses.^12^ The planimetric results also correlate well with other non-visual plaque assessment methods such as plaque weight and viable microbial counts.^94^

However, there are some drawbacks associated with planimetries too. These include inherent artifacts due to reflected light or discoloration of the acrylic denture base. Additionally, image quality consequential to the standardization of the camera settings such as the resolution power, the exposure time, as well as background lighting are all factors that need to be considered.^12^^,^^22^ Other factors that affect the image quality are the angulation between the camera and the denture,^25^^,^^29^^,^^69^, ^70^, ^71^^,^^76^ and the denture position^25^ and distance all of which should be standardized.

In conclusion, computerized planimetries provide a more objective assessment of denture plaque biofilm and do not require the calibration of assessors in comparison to naked eye visual assessment methods using denture plaque indices.^71^^,^^95^ Although time-consuming,^10^^,^^12^^,^^73^ and requires additional equipment as well as effort for capturing and analyzing standard images, some have suggested that planimetries should be the method of choice for research on denture hygiene.^71^^,^^73^ On the contrary, the naked eye assessment using denture plaque indices are simple, and easy to interpret though subjective,^22^ and perhaps more practical for those without access to imaging technology, providing acceptable results in a clinical setting. The latter, we believe, is more appropriate for community-level studies. Finally, very few plaque biofilm assessments of metallic denture bases have been conducted, and further work in this area is needed.

## Funding resources

This research/review did not receive any specific grant from funding agencies in the public, commercial, or not-for-profit sectors.

## Acknowledgements

Publication made possible in part by support from The University of Hong Kong (HKU) Libraries Open Access Author Fund sponsored by the HKU Libraries.

## CRediT authorship contribution statement

**Khaing Myat Thu:** Conceptualization, Methodology, Validation, Formal analysis, Investigation, Resources, Data curation, Writing – original draft, Writing – review & editing, Visualization. **Andy Wai Kan Yeung:** Conceptualization, Methodology, Validation, Formal analysis, Writing – review & editing, Supervision. **Lakshman Samaranayake:** Validation, Writing – review & editing, Supervision. **Walter Yu Hang Lam:** Conceptualization, Methodology, Validation, Writing – review & editing, Supervision, Project administration.

## Declaration of Competing Interest

The authors declare that they have no known competing financial interests or personal relationships that could have appeared to influence the work reported in this paper.

## Appendix Supplementary materials

Image, application 1

## Footnotes

Supplementary material associated with this article can be found in the online version at doi:10.1016/j.identj.2023.06.010.
