# Supplementary material for: Denture Plaque Biofilm Visual Assessment Methods: A Systematic Review
Source: Int Dent J. 2023 Jul 19;74(1):1–14. doi: 10.1016/j.identj.2023.06.010 (PMC10829380; doi:10.1016/j.identj.2023.06.010)
Supplement: Supplementary file 1 [file mmc1.docx]

| **No.** | **Year** | **Study** | **Type of study for respective quality assessment tools** | **National Institutes of Health (NIH) quality assessment tool questionnaires for respective type of study** | | | | | | | | | | | | | | **Total Y** |
| --- | --- | --- | --- | --- | --- | --- | --- | --- | --- | --- | --- | --- | --- | --- | --- | --- | --- | --- |
|  |  |  |  | Q1 | Q2 | Q3 | Q4 | Q5 | Q6 | Q7 | Q8 | Q9 | Q10 | Q11 | Q12 | Q13 | Q14 |  |
| **1** | 1970 | Budtz-Jorgensen and Bertram^4^ | Case control study | Y | N | N | Y | N | Y | - | Y | Y | Y | N | - |  |  | 6 |
| **2** | 1977 | Budtz-Jorgensen and Kelstrup^45^ | N-RCT | N | - | - | N | N | Y | Y | Y | Y | Y | Y | N | Y | - | 7 |
| **3** | 1977 | Budtz-Jorgensen^56^ | N-RCT | N | - | - | - | - | Y | Y | Y | Y | Y | Y | N | Y | - | 7 |
| **4** | 1978 | Budtz-Jorgensen and Knudsen^67^ | N-RCT | N | - | - | - | - | Y | Y | Y | Y | Y | Y | N | Y | - | 7 |
| **5** | 1981 | Abelson et al.^78^ | Observational cohort | Y | N | - | - | - | Y | - | - | Y | - | Y | N | - | - | 4 |
| **6** | 1982 | Ambjørnsen et al.^53^ | Observational cohort | Y | N | - | - | N | - | - | - | - | - | Y | N | - | - | 2 |
| **7** | 1982 | Ghalichebaf et al.^68^ | Case control study | Y | N | N | Y | - | Y | N | Y | Y | Y | N | - |  |  | 6 |
| **8** | 1982 | Tarbet et al.^83^ | Observational cohort | Y | N | - | - | N | - | - | - | - | - | Y | N | - | - | 2 |
| **9** | 1982 | Augsburger and Elahi^84^ | Observational cohort | Y | Y | Y | Y | N | - | - | - | - | Y | Y | N | - | - | 6 |
| **10** | 1983 | Budtz-Jorgensen et al. ^15^ | N-RCT | N | - | - | - | - | Y | Y | Y | Y | Y | Y | N | Y | - | 7 |
| **11** | 1983 | Poulsen et al.^16^ | Observational cohort | Y | N | Y | - | N | N | - | - | - | - | Y | N | - | - | 3 |
| **12** | 1984 | Ambjørnsen et al.^17^ | Observational cohort | Y | N | - | - | N | - | - | - | - | - | Y | N | - | - | 2 |
| **13** | 1986 | Murray et al.^18^ | Observational cohort | Y | Y | Y | Y | N | N | Y | Y | Y | - | Y | N | - | - | 8 |
| **14** | 1987 | Schou et al.^54^ | Cross-sectional study | Y | Y | Y | Y | N | - | - | - | - | - | Y | N | - | - | 5 |
| **15** | 1990 | Cardash et al.^19^ | Pre-Post study | Y | N | Y | Y | - | Y | Y | N | Y | Y | - | - |  |  | 8 |
| **16** | 1990 | Hoad-Reddick et al.^82^ | Cross-sectional study | Y | Y | Y | Y | N | N | - | - | - | - | Y | N | - | - | 5 |
| **17** | 1995 | McCabe et al.^20^ | Observational cohort | Y | N | - | - | N | - | - | - | - | - | Y | N | - | - | 2 |
| **18** | 1996 | Jeganathan et al.^21^ | Observational cohort | Y | Y | Y | Y | N | - | - | - | - | - | Y | N | - | - | 5 |
| **19** | 1996 | McCabe et al.^22^ | Observational cohort | Y | N | Y | - | N | Y | - | Y | Y | - | Y | Y | - | - | 7 |
| **20** | 1996 | Keng and Lim^23^ | Pre-Post study | Y | N | Y | N | - | Y | Y | N | - | Y | N | - |  |  | 5 |
| **21** | 1997 | Jeganathan et al.^24^ | N-RCT | N | - | - | N | N | Y | Y | Y | Y | Y | Y | N | Y | - | 7 |
| **22** | 2000 | Sheen and Harrison^25^ | RCT | Y | Y | N | N | Y | Y | Y | Y | Y | Y | Y | N | Y | Y | 11 |
| **23** | 2002 | Kulak-Ozkan et al.^26^ | Cross-sectional study | Y | Y | Y | - | N | - | - | - | - | - | Y | N | - | - | 4 |
| **24** | 2004 | Paranhos et al.^10^ | Observational cohort | Y | Y | Y | Y | N | - | - | - | - | - | Y | N | - | - | 5 |
| **25** | 2004 | Andrucioli et al.^69^ | N-RCT | N | - | N | N | N | Y | Y | Y | Y | Y | Y | N | Y | - | 7 |
| **26** | 2005 | Kanli et al.^27^ | Cross-sectional study | Y | Y | Y | Y | N | - | - | - | - | - | Y | N | - | - | 5 |
| **27** | 2006 | Montal et al.^55^ | Cross-sectional study | Y | Y | Y | Y | N | - | - | - | - | Y | Y | N | - | - | 6 |
| **28** | 2006 | De Visschere et al.^28^ | Cross-sectional study | Y | Y | Y | Y | N | Y | - | - | Y | - | Y | - | - | - | 7 |
| **29** | 2006 | Dikbas et al.^57^ | Cross-sectional study | Y | Y | Y | Y | N | - | - | - | - | - | Y | N | Y | - | 6 |
| **30** | 2007 | Fernandes et al.^70^ | Case control study | Y | N | N | Y | N | Y | - | Y | - | Y | N | - |  |  | 5 |
| **31** | 2007 | Paranhos et al.^29^ | Pre-Post study | Y | Y | Y | Y | N | Y | Y | N | Y | Y | - | - |  |  | 8 |
| **32** | 2007 | Paranhos et al.^30^ | Observational cohort | Y | Y | Y | Y | N | - | - | - | - | - | Y | N | - | - | 5 |
| **33** | 2007 | Salles et al.^71^ | Observational cohort | Y | N | - | Y | N | Y | Y | Y | Y | Y | Y | N | - | - | 8 |
| **34** | 2009 | Coulthwaite et al.^12^ | N-RCT | N | - | N | - | - | Y | - | - | Y | Y | Y | - | Y | - | 5 |
| **35** | 2010 | Paranhos  et al.^31^ | Observational cohort | Y | Y | Y | Y | N | N | N | N | N | N | Y | N | - | - | 5 |
| **36** | 2010 | Souza et al.^72^ | RCT | Y | Y | N | N | Y | Y | - | - | Y | - | Y | N | Y | - | 7 |
| **37** | 2011 | Cruz et al.^73^ | N-RCT | N | Y | - | N | Y | Y | Y | Y | Y | Y | Y | N | Y | - | 9 |
| **38** | 2012 | Puskar et al.^58^ | Cross-sectional study | Y | Y | Y | Y | N | - | - | - | - | - | Y | N | - | - | 5 |
| **39** | 2012 | Taiwo et al.^59^ | Cross-sectional study | Y | Y | Y | Y | N | N | - | - | - | - | Y | N | - | - | 5 |
| **40** | 2012 | de Andrade et al.^74^ | N-RCT | N | Y | - | N | Y | Y | Y | Y | Y | Y | Y | N | Y | - | 9 |
| **41** | 2013 | Sloane et al.^32^ | Pre-Post study | Y | Y | Y | Y | - | Y | Y | N | Y | Y | - | - |  |  | 8 |
| **42** | 2014 | Mylonas et al.^33^ | Retrospective cohort | Y | Y | Y | Y | N | - | - | - | - | - | Y | N | - | - | 5 |
| **43** | 2014 | Zenthöfer et al.^34^ | Observational cohort | Y | Y | Y | Y | N | Y | - | Y | Y | - | Y | N | - | - | 8 |
| **44** | 2014 | Zenthöfer et al.^35^ | Observational cohort | Y | Y | N | Y | N | N | - | - | - | - | Y | N | - | - | 4 |
| **45** | 2015 | Almas et al.^36^ | Cross-sectional study | Y | Y | Y | Y | N | Y | - | - | - | N | - | N | - | - | 5 |
| **46** | 2015 | Khanagar et al.^37^ | Cross-sectional study | Y | Y | Y | Y | N | - | - | - | - | - | Y | N | - | - | 5 |
| **47** | 2015 | Zenthöfer et al.^38^ | N-RCT | N | - | N | N | N | N | Y | Y | Y | Y | Y | N | Y | - | 6 |
| **48** | 2016 | Mylonas et al.^39^ | Cross-sectional study | Y | Y | Y | Y | - | - | - | - | - | - | Y | N | - | - | 5 |
| **49** | 2016 | Steinmassl et al.^65^ | Cross-sectional study | Y | Y | Y | Y | N | - | - | - | Y | - | Y | N | - | - | 6 |
| **50** | 2016 | Zenthöfer et al.^40^ | Observational cohort | Y | Y | Y | Y | N | N | Y | - | Y | N | Y | N | - | - | 7 |
| **51** | 2016 | Duyck et al.^41^ | RCT | Y | Y | - | - | - | Y | Y | Y | Y | Y | Y | Y | Y | Y | 11 |
| **52** | 2016 | Al-Kaisy et al.^75^ | Cross-sectional study | Y | Y | Y | Y | N | - | - | - | - | - | Y | Y | - | - | 6 |
| **53** | 2016 | Zenthöfer et al.^42^ | Case control study | Y | Y | N | Y | N | Y | - | Y | Y | Y | N | - |  |  | 7 |
| **54** | 2017 | Nihtila et al.^60^ | Case control study | Y | Y | N | Y | Y | N | Y | Y | Y | Y | N | Y |  |  | 9 |
| **55** | 2017 | Martori et al.^43^ | Cross-sectional study | Y | Y | Y | Y | N | - | - | - | - | - | Y | Y | - | - | 6 |
| **56** | 2017 | Zimmerman et al.^44^ | Cross-sectional study | Y | Y | Y | Y | N | - | - | - | - | - | Y | Y | - | - | 6 |
| **57** | 2017 | Arruda et al.^76^ | RCT | Y | Y | Y | N | N | Y | Y | Y | Y | Y | Y | N | Y | Y | 11 |
| **58** | 2018 | Baba et al.^77^ | N-RCT | N | Y | - | N | N | Y | Y | Y | Y | Y | Y | N | Y | Y | 9 |
| **59** | 2018 | Ikeya et al.^79^ | N-RCT | N | - | - | N | N | Y | Y | Y | Y | Y | Y | N | Y | - | 7 |
| **60** | 2018 | Schwindling et al.^46^ | N-RCT | N | - | N | N | N | Y | N | N | Y | Y | Y | N | Y | - | 5 |
| **61** | 2018 | Klotz et al.^47^ | Case control study | Y | Y | N | Y | Y | Y | - | Y | Y | Y | N | Y |  |  | 9 |
| **62** | 2018 | Guevara-Canales et al.^61^ | Cross-sectional study | Y | Y | Y | Y | N | - | Y | - | Y | - | Y | N | - | Y | 8 |
| **63** | 2018 | Weintraub et al.^48^ | RCT | Y | - | - | - | - | Y | N | N | Y | Y | Y | N | Y | Y | 7 |
| **64** | 2020 | Klotz et al.^49^ | Observational cohort | Y | Y | Y | Y | Y | Y | Y | - | Y | N | Y | N | Y | Y | 11 |
| **65** | 2020 | Badaró et al.^80^ | RCT | Y | Y | Y | Y | Y | Y | Y | Y | Y | Y | Y | Y | Y | Y | 14 |
| **66** | 2020 | Krausch-Hofmann et al.^11^ | N-RCT | N | - | - | - | - | N | - | - | Y | Y | Y | N | Y | - | 4 |
| **67** | 2021 | Alqarni et al.^50^ | Case control study | Y | Y | N | Y | Y | Y | Y | Y | Y | Y | Y | Y | N | - | 11 |
| **68** | 2021 | Grag et al.^51^ | Observational cohort | Y | Y | Y | Y | N | Y | Y | Y | Y | N | Y | N | Y | Y | 11 |
| **69** | 2021 | Ng et al.^52^ | RCT | Y | Y | N | N | N | Y | Y | Y | Y | - | Y | Y | Y | - | 9 |
| **70** | 2021 | Cinquanta et al.^52^ | Cross-sectional study | Y | Y | Y | Y | Y | N | - | - | - | - | Y | N | - | - | 6 |
| **71** | 2021 | Wiatrak et al.^66^ | Case control study | Y | Y | N | Y | - | Y | - | Y | - | Y | N | - |  |  | 6 |
| **72** | 2021 | Araujo et al.^81^ | RCT | Y | Y | Y | Y | Y | Y | Y | Y | Y | Y | Y | Y | Y | Y | 14 |
| **73** | 2022 | Mousa et al.^63^ | Case series | Y | Y | Y | Y | - | Y | - | Y | Y |  |  |  |  |  | 7 |
| **74** | 2022 | Peroz et al.^64^ | Pre-Post study | Y | Y | Y | Y | N | Y | Y | N | N | Y | - | - |  |  | 7 |

Supplementary Table 1 Risk of bias assessment using National Institutes of Health (NIH) quality assessment tool according to type of studies (Y=Yes, N=No, (-)=Not Accessible, RCT=Randomized controlled trial, N-RCT=Non-Randomized Controlled Trial)
